# Excessive Exercise—A Meta-Review

**DOI:** 10.3389/fpsyt.2020.521572

**Published:** 2020-11-20

**Authors:** Flora Colledge, Robyn Cody, Ursula G. Buchner, André Schmidt, Uwe Pühse, Markus Gerber, Gerhard Wiesbeck, Undine E. Lang, Marc Walter

**Affiliations:** ^1^Department of Sport, Exercise and Health, University of Basel, Basel, Switzerland; ^2^Deutsche Hochschule für Gesundheit und Sport GmbH, Ismaning, Germany; ^3^University Psychiatric Clinics, University of Basel, Basel, Switzerland

**Keywords:** exercise addiction, behavioral addiction, review, symptomatology, diagnostic criteria, diagnosis

## Abstract

**Background and Aims:** While a number of studies have reported on individuals who exercise excessively, and feel unable to stop despite negative consequences, there is still insufficient evidence to categorize exercise as an addictive disorder. The aim of this meta-review is to summarize the published articles and to compile a list of symptoms reported in the qualitative literature in conjunction with excessive exercise. This list is compared with the DSM-5 criteria for gambling disorder, and initial diagnostic criteria for exercise addiction are suggested.

**Methods:** The databases MEDLINE, Web of Science and PsycInfo were searched for qualitative studies or case reports, in which excessive exercise was the main focus. All symptoms reported in conjunction with excessive exercise were extracted from each study and documented. Symptoms were also compared to the diagnostic criteria for gambling disorder.

**Results:** Seventeen studies were included in the review, yielding 56 distinct symptoms. The Critical Appraisal Skills Program tool showed that the majority of the studies were of acceptable quality. Exercise-related symptoms corresponded with seven of the nine DSM-5 criteria for gambling disorder. The ten suggested criteria for exercise addiction are: increasing volume, negative affect, inability to reduce, preoccupation, exercise as coping, continuation despite illness/injury, minimization, jeopardized relationships, continuation despite recognizing consequences, guilt when exercise is missed.

**Discussion:** Our results suggest that excessive exercise may constitute a behavioral addiction, based on the criteria of the DSM-5.

**Conclusions:** Subsequent studies should aim to systematically classify symptoms of excessive exercise; in addition, it should be noted that basic questionnaires may be need to be supplemented with detailed clinical examinations.

## Introduction

### First Studies

The fact that the typically healthy practice of exercise may in some cases become unhealthy has been discussed in the scientific literature for almost 50 years. Since Baekeland (1) reported that subjects recruited to a study on sleep were highly resistant to changing their exercise habits to meet his requirements, exercise has come to be seen as a potentially negative pursuit. Due to the fact that the characteristics of harmful exercise habits seem, at face value, to have much in common with substance-related disorders, the term “exercise addiction” has been employed since the late 70 s (2). These characteristics, which in subsequent years have been cataloged in a number of case studies, include escalation, tolerance, continuance despite acknowledged negative effects, social conflict and withdrawal symptoms (3, 4). Ostensibly affected individuals spend several hours per day exercising (5, 6), prioritize exercise sessions above everything else in their lives (7), and feel unable to stop this pattern (8, 9). Individuals who stop experience anguish and guilt, and generally resume the habit very soon. These symptoms represent a psychological burden on this affected individual, but also frequently entail physical or health impairments, such as injury, poor recovery from injury, and chronic upper respiratory infection (10).

In more recent years, prevalence estimates have been made, although these have varied widely (11). For instance, figures of between 40 and 50% have been reported for some exercising populations, while in others, prevalence has been reported at below 10%; in studies of general populations, figures tend to be below 1% (12). This variance has been attributed in part to the variety of measurement instruments which are used to asses risk for a potential exercise addiction. Certain questionnaires contain items which may be unsuitable for distinguishing professional or amateur athletes from those who feel compelled to exercise (13). As little is currently known about the etiology of an addiction to exercise, the questionnaires are currently based on theoretical conceptualizations, rather than well-established disease histories. To date, reviews have reported a higher prevalence among men (14), and players of team sports appear to be affected as frequently as individual athletes (though more research has been carried out with the latter group) (15). Between 38 and 45% of individuals with eating disorders are also reported to suffer from symptoms of a possible exercise addiction (12). This high prevalence has led some scholars to suggest that symptoms which are attributed to “exercise addiction” are in fact only manifestations of an eating disorder, and should not be understood in the context of a separate behavioral addiction (16). However, most scholars accept that there is also evidence of individuals who exercise excessively without concerns about weight or food consumption; this distinction is addressed in more detail in section Primary and Secondary Exercise Addiction, below.

### Inclusion in the DSM

Currently, “exercise addiction” has not been universally accepted as a form of behavioral addiction (17, 18). To date, only gambling disorder is included in the DSM-5 as a non-substance-related disorder. The criteria for diagnosis resemble those for substance-related disorders (such as the development of a tolerance for the amount of time spent gambling and a corresponding need to increase it, continuance despite recognition of the negative consequences entailed by gambling, and feelings of anxiety and compulsion to continue when abstaining) but have some behavior-specific variations, such as the criteria referring to the urge to gamble to offset a “losing streak.” It has frequently been noted that the establishment of further behavioral addictions will involve similar tailored criteria (17). Broad theoretical frameworks for behavioral addictions in general have been proposed. The I-PACE model lays out a large number of variables which interact to foster a behavioral addiction, including individual characteristics, inhibitory control, and cue-reactivity (19). The core component of “gratification” can be fulfilled by a wide variety of behaviors or substances, and the model has gained widespread use in addiction research. A further component group, the specific needs, motives, and values of the individual, can also be adapted to specific behaviors. Consequently, the model does much to escape the criticism of some behavioral addiction research, namely that it is atheoretical and therefore owes too much to confirmatory studies of largely anecdotal evidence (18). The model also avoids including DSM criteria such as tolerance and withdrawal, which it has been argued do not have a place in behavioral addictions (20). Diagnostic criteria for exercise addiction can therefore be expected to resemble those of substance-related or gambling disorders, as certain key components are common to addictive disorders per se, but will include certain exercise-specific formulations or unique symptoms.

Partly as a consequence of the lack of conceptual work underpinning a possible exercise addiction, the phenomenon is not yet categorized as a non-substance related disorder in the DSM-5, nor recognized in the ICD 11. The American Psychological Association explicitly states that, as yet, there is insufficient evidence to definitively categorize the disorder, if it really is a disorder (21). However, a search of the PubMed database using the above-mentioned terms reveals that over 100 studies have addressed this phenomenon (13). A final key issue, therefore, is that these studies have not yet supplied enough information about core aspects of a potential exercise addiction.

### Primary and Secondary Exercise Addiction

A second theoretical consideration, specific to exercise behaviors, is the posited existence of “primary” and “secondary” forms of exercise addiction (22). This distinction, introduced by De Coverley Veale in 1987, takes as its central focus the motivation behind the exercise behavior. “Primary exercise dependence” should only be considered after an eating disorder (specifically anorexia or bulimia nervosa) has been excluded. In this case, the motivation of the exerciser is not solely weight control, but pursuing exercise as an end in itself. In the presence of an eating disorder, the exercise behavior is understood as a means to an end, and is thus classed as “secondary.”

In summary, suspicions that exercise might constitute a behavioral addiction have not yet been backed up by strong evidence. While many avenues of research are required before a potential disorder can be fully characterized, a first step is to systematically synthesize what has to date been reported about the issue. It is also crucial to outline a possible path for future clinical interviews.

### Aims

In this article, we chose not to use the term “exercise addiction,” as this would be akin to assuming a fact that we are trying to investigate. The same is true for terms such as “compulsive” or “obligatory” exercise, which imply psychological dimensions of the phenomenon that have not yet been satisfactorily demonstrated. We have used the term “excessive exercise,” without quotation marks from this point on, as this captures the necessary criterion of exercise going beyond with is healthy (or, in athletes, required) without invoking terminology from the psychological lexicon.

The primary aim of this study was to compile an exhaustive list of symptoms that have been associated with excessive exercise. Due to the limitations of questionnaire-based research in this field, noted above, and the lack of detailed symptom description in such studies (as respondents can only rate a standard list of statements, rather than detail their own experiences), this meta-review focused solely on case reports and clinical interviews. This pool of data presents an excellent source for examining symptoms which have been reported in the context of excessive exercise, as detailed feedback from study participants can be reported verbatim. To date, this data has never been synthesized. We have compared the resulting list of symptoms with the DSM-5 criteria for gambling disorder, in order to illustrate whether there are parallels which may inform the future categorization of excessive exercise. Furthermore, we have presented an initial suggestion for 10 criteria to be used in a clinical interview screening for possible “exercise addiction.”

The secondary aim was to identify any inconsistencies between questionnaire-based and qualitative investigations of excessive exercise, in qualitative studies which also employ quantitative measures. This is to enable an assessment of whether currently employed questionnaires are useful in assessing unhealthy exercise behavior.

This review is important because reports of excessive exercise indicate that affected individuals not only suffer from a psychological burden comparable to that of other behavioral and substance-related addictions, but also because excessive exercise can have a negative impact on physical health. Studies report individuals training despite broken bones and severe muscular injury; furthermore, overtraining syndrome, which severely impacts the health of affected individuals, may also originate with excessive exercise. Diagnostic criteria for exercise addiction will therefore be an important step in identifying and offering treatment to those affected.

## Materials and Methods

The current study was carried out following the guidelines of the Preferred Reporting Items for Systematic Reviews and Meta-Analyses (PRISMA) (23).

### Meta-Review

**Figure 1:**
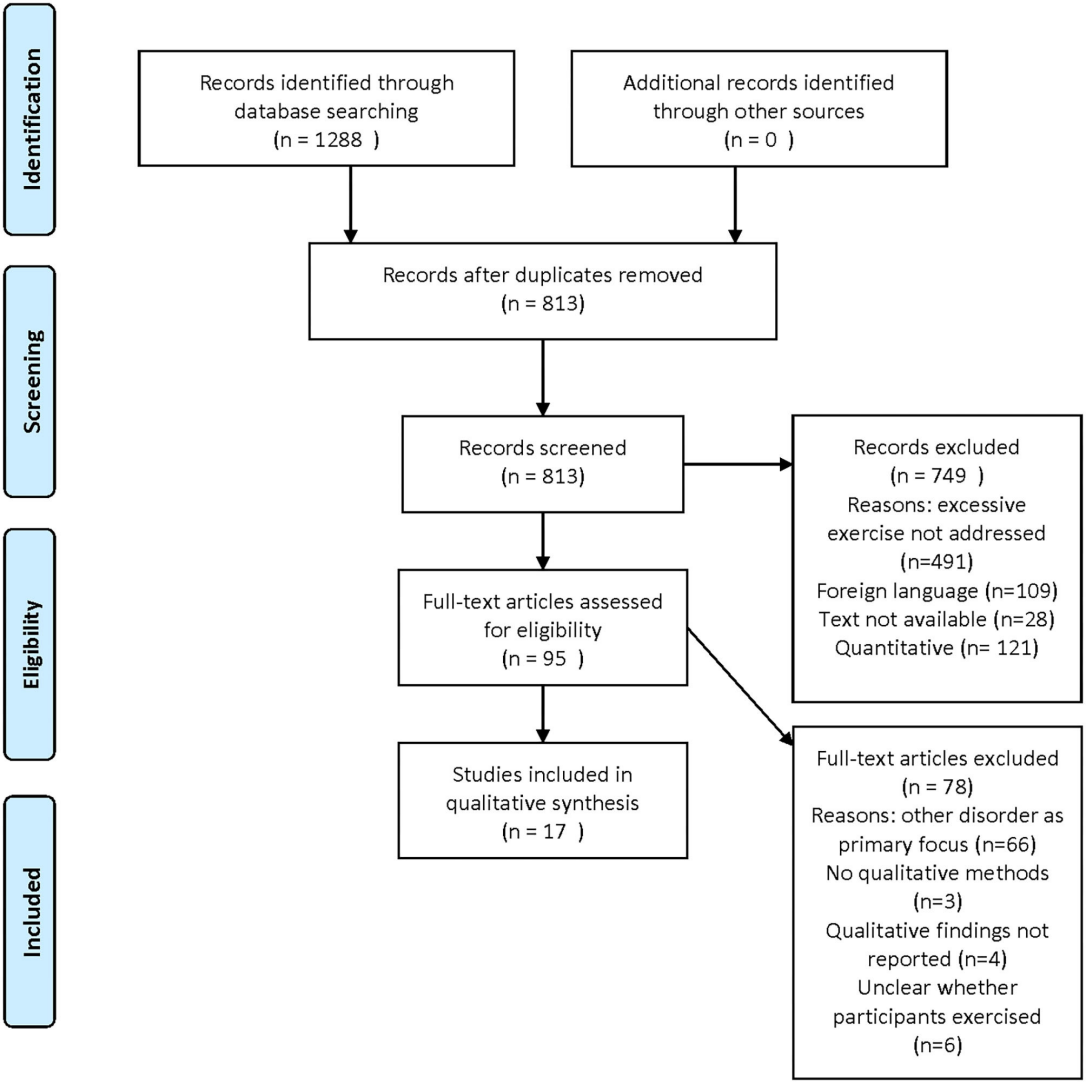
PRISMA flow diagram.

**Figure 2:**
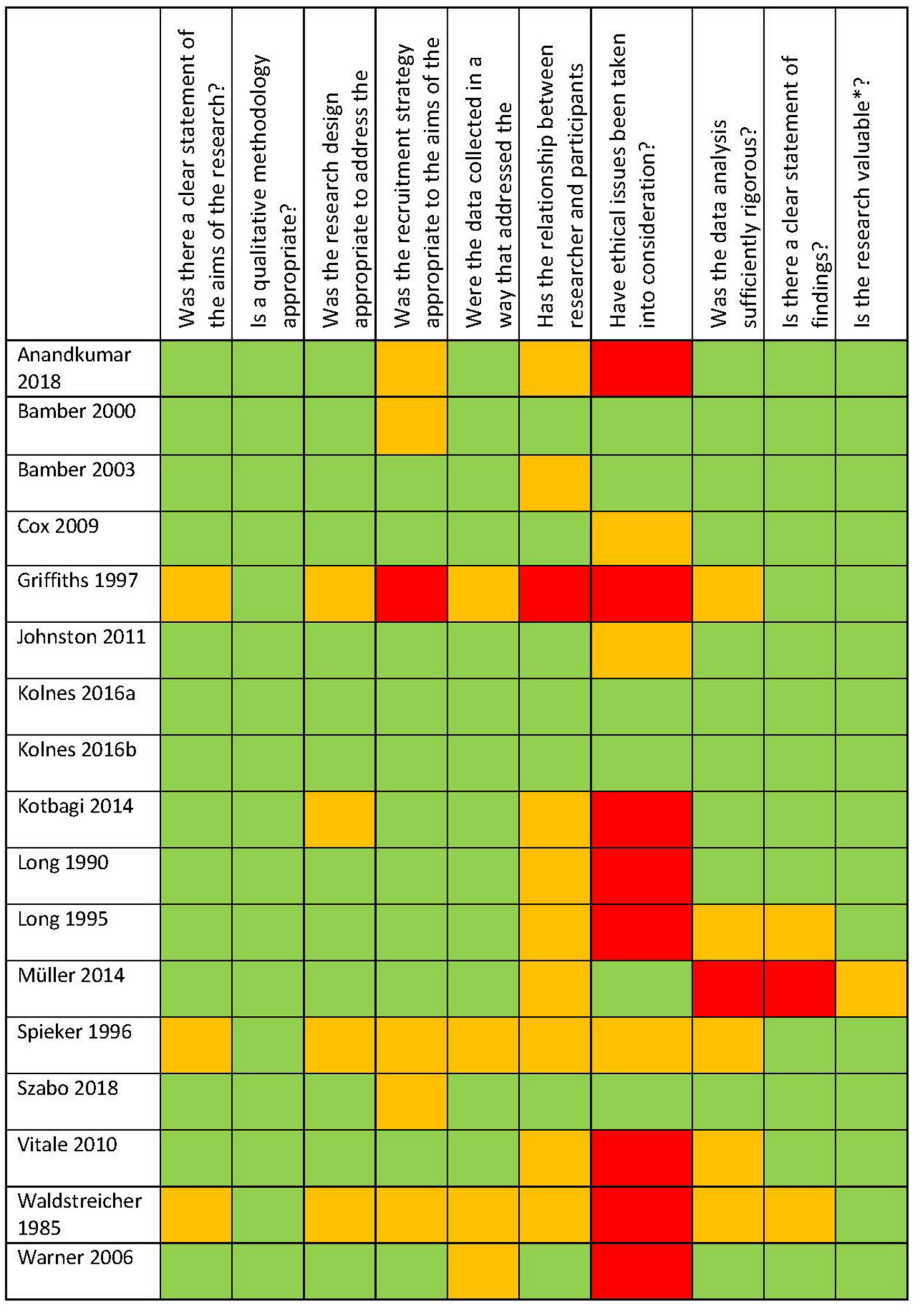
CASP quality assessment.

A meta-review is a systematic extraction and synthesis of qualitative research findings. This method is suited to providing a description of a new phenomenon (24). As noted above, numerous quantitative studies have examined the phenomenon of excessive exercise. However, these studies do not involve asking affected individuals about their symptoms, and are therefore not suited to addressing a research aim that involves cataloging symptoms. We have therefore focused solely on qualitative studies, case reports, and resources which involve, by their nature, a description of the symptoms experienced. For this reason, numerous studies on excessive exercise have been excluded if they contain only quantitative data (see Figure 1), and the included studies are sometimes of poor quality (see Figure 2) or not peer-reviewed. The decision to include them was based on prioritizing the exhaustiveness of the search. However, the reader must be aware that such studies are presented here. While our approach is different from a traditional meta-analysis, and excludes a number of studies addressing the topic of excessive exercise, this study uniquely targets the experience of excessive exercise.

### Search Strategy

The MEDLINE, Web of Science and PsycINFO databases were searched. All studies from the date of database creation until the 10th of January 2019 were considered eligible. The following terms were used: “exercise addiction” or “exercise dependence” or “compulsive exercise” or “obsessive exercise” or “obligatory exercise” or “over-exercise” or “excessive exercise” AND “qualitative” or “interview” or “case.” While the terms were only entered in English, studies in English, French or German were included if they were identified following these methods. Manual searches of the reference list of articles identified as being relevant were also carried out. The titles and abstracts of retrieved studies were read, independently, by two team members (FC and RC). Studies meeting the inclusion criteria were gathered, and compared by the two researchers. Any discrepancies were discussed, and in case of uncertainty, were determined by a third team member (MW). The final list of studies included in the review is the result of this process.

#### Inclusion and Exclusion Criteria

Studies which met the following criteria were included in the current meta-review: (a) high levels of physical activity or exercise, which are associated with psychological distress for the sufferer in some way, represent the main psychiatric issue, phenomenon or disorder under discussion, irrespective of the name ascribed to that issue; (b) qualitative interviewing or a case description was amongst the methods used. Qualitative studies including quantitative measure were therefore considered suitable; quantitative studies with no qualitative assessment were not.

The following exclusion criteria were applied: A disorder other than excessive exercise was the condition under examination, even though participants might have excessive exercise habits.

### Quality Assessment

Following Lachal et al. (25) summary of possible approaches to study quality evaluation in meta-reviews, we employed the Critical Appraisal Skills Program (CASP) tool (Critical Appraisal Skills Programme, 2018) for qualitative research. This simple checklist allows for a number of quality indicators relevant for qualitative studies to be categorized as totally met, partially met, or not met. The tool is among those recommended by the Cochrane Collaboration for the evaluation of qualitative studies. There is not standard cut-off value for this checklist. In view of the fact that we are seeking to summarize all available evidence related to our topic, we have not excluded any study based on poor quality. However, an overview of study quality may be useful in future research on this topic, so we have included the CASP checklist in Figure 2.

### Data Extraction

FC and RC independently read all included studies in full. The following parameters were extracted for this meta-review: study methodology, number of participants, participant characteristics, forms of exercise engaged in by participants, the volume (in hours) of training engaged in by study participants, problematic symptoms reported as a result of exercise habits, inconsistency between qualitative and quantitative findings, psychiatric comorbidities of the participants (at any time).

The comparison with DSM-5 criteria for gambling disorder was achieved by FC and MW independently agreeing on the symptoms from the initial list of 57 which best fit the criteria (in some cases, more than one symptom is matched to one criterion). The suggested criteria for a clinical interview were arrived independently by FC and MW, and agreed upon with RC. This two-step process involved first compiling all severe psychologically burdensome symptoms from the initial list of 57, and organizing these in line with the DSM criteria for addictive disorders. These are *not* to be understood as criteria for inclusion in the DSM framework at this stage, or an attempt to map exercise habits onto the current framework. Rather, this is a guide for clinical interviewing to support the establishment of evidence-based criteria in the future.

## Results

The PRISMA flow diagram in Figure 1 shows the number of studies identified, screened and included. Following screening, 749 articles were excluded as they were not relevant to the question addressed here. Reasons for further exclusions are presented in Figure 1.

### Study Characteristics

Seventeen studies (5–9, 26–37) were included in this meta-review. Sample sizes ranged between 1 (5, 6, 9, 31, 33, 35, 36) and 134 (34). This comprised seven case descriptions (5, 6, 9, 26, 31, 33, 36), six qualitative semi-structured interviews (7, 8, 27–30) (of which two are in fact separate analyses of a single data pool), one qualitative structured interview (34), one written questionnaire with open questions (37), and two qualitative interviews of unclear form (32, 35). Nine also employed quantitative instruments to assess problems related to exercise (7, 26–29, 31, 34, 35, 37). For the following section on study characteristics, the two articles which report on a single data pool (8, 30) were only be counted once (although both citations have been listed).

Fourteen studies reported on the general form of exercise engaged in by the participants (5–9, 26–31, 33, 35–37). In five studies, participants from a variety of exercise forms were represented (8, 27–30, 38). Among all studies, individual exercise was reported in 13 cases (6–9, 26–31, 33, 35–37), competitive sport participation in seven cases (5, 7, 8, 27, 29, 30, 39), professional sporting activity in 3 cases (7, 28, 29), and team sport participation in one case (29).

Ten studies reported the approximate volume (in hours) of exercise engaged in by their participants (7–9, 26, 28–31, 33, 35). Hours per week range from 1 (29) to 24 (28) (this includes figures from studies involving individuals who do not exercise excessively).

Ten studies reported that at least some respondents had been diagnosed with, or met the diagnosis criteria for, a psychiatric comorbidity (6–8, 27, 29–34, 36). In all but one case (31), the diagnosis was an eating disorder, and in six (7, 8, 27, 30, 33, 34), participants were at least partially recruited through eating disorder treatment centers or organizations. This is an important potential confounder for this study, as a number of symptoms listed here may in fact result directly and solely from eating disorders. We have included these studies, because, as stated above, they examine exercise as the primary problematic behavior. In our symptom list, we also highlighted all symptoms identified in studies where a comorbidity was diagnosed (*n* = 36).

Six studies reported some degree of inconsistency between the various measurements of excessive exercise employed (7, 27–29, 34, 35). Typically, this is between qualitative and quantitative instruments.

All studies and extracted data are presented in Table 1.

**Table 1:** Data extracted from included studies.

**References**	**Type**	***N* (total)**	**Subjects (recruitment strategy)**	**Exercise types**	**Volume (time) engaged in by participants**	**Symptoms**	**Inconsistency**	**Psychiatric comorbidities**
Waldstreicher (36)	Case description (letter to editor)	1	Female (presenting to MD with hip pain)	Individual	2–3 h/day	- High volume - Not underweight - Low calorie intake - Refusal to admit behavior to treating physician	-	Anorexia
Long and Smith (33)	Case description	1	Female (ED inpatient)	Individual	2–6 h/day	- High volume - Rumination - Would do more if possible - Tension, frustration and anxiety if stopped - Food adjusted as compensation - Motivation: control mood, self-esteem, fitness - Recognition of excess and neglect of goals	-	Anorexia, bulimia, laxative abuse
Long (32)	Interview (form unclear)	6	Former ED inpatients	-	-	- Exercise motivated by body shape - Exercise as a means of regulating affect - Possible dread of exercise - Uncertain whether exercise is enjoyed - Change of exercise after intervention attempting to decrease it - Compulsive	-	Anorexia, bulimia, EDNOS
Spieker (6)	Case description	1	Female (presenting to MD with hip pain)	Individual	-	- Feeling good (during? after?) - Escalation - Training through injury - High volume training immediately following injury	-	Previous anorexia and alcohol addiction Suicide attempt Current depression
Griffiths (5)	Case description	1	Female (psychiatric outpatient)	Competitive, martial art	-	- High volume - Primary importance - Conflict with study - Increased volume over time - Agitation and irritability when session missed - Other tasks only possible after exercise - Feeling high - Social conflict - Unable to stop despite attempts - Spending beyond means (socially unacceptable methods of earning money) - Training through injury	-	-
Bamber et al. (7)	Quantitative screening (EXDI); 2-part semi-structured interview	16 (4 primary ExD, 4 secondary ExD, 2 ED, 3 control)	Females (dance classes, sport centers, university sport clubs, running clubs, Eating Disorder Association, members, psychiatrist refs, ED patients, running magazine readers, members of UK Athletics Org.	Competitive, individual, professional	Up to 4 h/day	- Feeling unable to stop - Exercise for performance/weight (weightbearing sport) - Training through illness/injury - Social life conflict - Wishing to stop but unable (fear of mood and weight) - Volume (many hours/beyond training requirements) - Structured - Left job - Controls all aspects of life - Withdrawal (tension, agitation, insomnia, depression, headache, suicidal ideation, anger, lethargy) - Exercise as painful punishment	Y	Eating disorder
Bamber et al. (27)	Quantitative assessment (EDQ), semi-structured interview	16	Females (aerobics classes, sport centers, athletics clubs, Eating Disorder Association, running magazine readers	Competitive, individual	-	- Impaired functioning (concentration) - Social conflict - Leaving social situation to exercise - Inability to concentrate	Y	Eating disorder
Warner and Griffiths (37)	Quantitative assessment (EAI) + 4 written open questions	100	Mix (gym users)	Individual	**-**	**Above EAI cutoff only** - Weight and body image motivations - Improved self-esteem motivation - Anger at injury, exacerbating injury by continued training - Stress relief - Self-confessed addiction - Exercise most important thing in life - Escalation to feel escapism or high social conflict - Return after a period of abstinence	N	-
Cox and Orford (28)	Quantitative assessment (EDQ), semi-structured interview	10	Mix (gyms snowballing among exercisers)	Individual, professional, martial arts	7–24 h/week	- Gaining control - Stress relief - Feeling better while exercising - Able to control volume - Enjoying structure of led classes - Exercise as reliable comfort - Desire for an ideal physique - Fear of losing physique if exercise stopped - Feeling of enjoyment		
						- Exercising through illness and injury, regardless of medical opinion - Unwillingness to stop due to fear of low mood, depression - Feelings of guilt and unfitness when stopping training - Accepting possible future negative consequences - Satisfaction when completed - Having and meeting goals (delusion?)	Y	- (anorexia explicit exclusion criterion)
Vitale et al. (9)	Case description and *post-hoc* DSM 4 substance dependence symptom comparison	1	Male (treatment for Parkinson's disease)	Individual	~3 h/day	- High volume - Escalation - Feeling of well-being (“achieved” by exercising) - Unable to reduce - Withdrawal (anxiety, irritation, depression) (ended following removal of pramipexole and introduction of levodopa)	N	-
Johnston et al. (29)	Quantitative assessment (OEQ), semi-structured interview	32	White females (exercisers, weight loss groups, school and university, word of mouth)	Competitive, team, individual, professional	1–16 h/week (*M* = 5)	- Exercise fits around other things - Solution to problems - Compensation/replacement for disturbed eating - Routine leading to rigidity - Routine leading to increased withdrawal anxiety - Not always concurrent with disturbed eating	Y	Possible disordered eating
Kotbagi et al. (31)	Quantitative assessment (EDS-R EDQ), case description	1	Male (self-referral to psychiatric services)	Individual, former successful amateur competitor	10 h/week	- Social conflict - Shift work to accommodate training - Rigid structure over 15 years - Financial cost of participation - Need for escalation to feel good - Tension when forced to stop	N	Previous body dissatisfaction, compulsive shopping, sex addiction
Müller et al. (34)	Quantitative assessment (EDS), selected structured interviews	134	Gym goers, ED patients, sport students, hobby athletes	-	-	NO DATA	Y	Bulimia, Anorexia
Kolnes (8)	Semi-structured interview	6	Female ED patients, 4 former athletes	Individual, competitive	2–3 h/day	- Feeling of compulsion - Reducing social interactions (lying) - Minimizing impact - Minimizing volume - Increase over time		
						- Unable to stop despite health consequences - Personal rules (minimum distance run, even numbers) - Training through illness - Training beyond prescribed schedule - Torture/boredom - Persistence despite rational understanding of the consequences - Exercise during routine activities - Exercise to compensate for ED therapy - Exercise has primary importance in life - Need to feel control	N	Anorexia
Kolnes Rodriguez-Morales (30)	See Kolnes (8)	See Kolnes (8)	See Kolnes (8)	See Kolnes (8)	See Kolnes (8)	- Emotion regulation - Disgust with the body	See Kolnes (8)	See Kolnes (8)
Szabo et al. (39)	Quantitative assessment (EAI; to be assessed qualitatively) Interview (form unclear)	1	Female (campus-wide search, exercise addiction symptoms)	Competitive, individual	From 7.5 h/week	- Training beyond prescription due to guilt - Positive attitude to appearance change - Prioritized over social life but concerned about this - Dislike of strict diet - Workplace and social conflict	Y	-
Anandkumar et al. (26)	Quantitative assessment (EAI), case description	2	Male (back pain)	Individual	6 h/week	- Feeling a high - Control over site of injury - Tired, guilty, anxious when session missed - Relief of injury pain through exercise - Thoughts constantly turning to exercise - Anguish when unable to exercise while working	N	-

*MD, medical doctor; ED, eating disorder; EDNOS, eating disorder not otherwise specified; EXDI, Exercise Dependence Interview; ExD, exercise dependence; EDQ, Exercise Dependence Questionnaire; EAI, Exercise Addiction Inventory; OEQ, Obligatory Exercise Questionnaire; EDS-R, Exercise Dependence Scale–Revised; EDS, Exercise Dependence Scale*.

### Symptoms

Table 2 provides an exhaustive list of symptoms described in the included studies (*n* = 56). These symptoms were reported as being consequences of the participants' exercise habits; however, in a number of cases, the participants also report other psychiatric disorders, so an overlap of symptoms from, for example, eating disorders cannot be excluded. For this reason, we have marked all symptoms reported in a study including other comorbidities with an asterisk.

**Table 2:** Symptoms reported as emerging in individuals who exercise excessively (*n* = 56).

Body	• Exercise is used to control weight for athletic performance^*^ • The individual's body is a source of disgust^*^ • Enjoyment of appearance change elicited by exercise • Preoccupation with physical appearance and /or muscles • Fear of losing physique if exercise habits are altered • Idealized future when physical goals are attained • Exercise is motivated by weight/body image concerns
Food	• Exercise is used as a compensatory behavior in disturbed eating, or as an alternative to restriction/preferred method^*^ • Persistently low calorie intake^*^ • Food intake reduced to compensate if training is missed^*^
Negative Experiences	• Withdrawal symptoms are feared • Exercise is continued through illness/injury, and/or contrary to medical advice^*^ • Exercise habits cause conflicts in social relationships^*^ • Exercise causes difficulties in carrying out work/study responsibilities, may lead to reducing working hours/leaving job/studies • Exercise is used as self-punishment^*^ • Exercise is perceived negatively (boring, tortuous)^*^ • Persistence despite rational understanding of negative impact/future likely negative impact • Guilt when exercise is insufficient • Cognitive functioning is impaired^*^ • Thoughts constantly turning to exercise/rumination^*^ • Lying about/hiding exercise habits^*^ • Anger at injury • Spending beyond means on exercise-related items
Positive Experiences	• Exercise is seen as a way to solve/deal with problems and stress^*^ • Feeling “high” during/after exercise • Relief of injury pain through exercise • Feeling better during exercise • Exercise as a predictable comfort • Enjoyment of structure
Control	• The affected person feels unable to stop/ is unable to stop despite attempts^*^ • The affected person wishes they could stop^*^ • Individual feels compelled to exercise^*^ • Sense of control over body (also when injured) and life^*^
Withdrawal symptoms	• Tension,^*^ • Agitation,^*^ • Insomnia,^*^ • Depression,^*^ • Headache,^*^ • Suicidal ideation,^*^ • Anger,^*^ • Lethargy,^*^ • Anxiety,^*^ • Irritation, • Frustration^*^
Other characteristics	• Other things in life must fit around exercise • The structure of exercise undertaken is rigid^*^ • Exercise volume is high (between 2 and 7+ h daily)^*^ • The negative impact and high volume of exercise is minimized by the individual^*^ • Exercise volume has increased over time^*^ • Personal rules/superstitions govern behavior^*^
	• Coached athletes train beyond what is prescribed by trainer^*^ • Exercise is undertaken in the course of routine activities (e.g., pushups at work)^*^ • Exercise is judged to have primary importance in life^*^ • Rapid return to high exercise volume following a period of abstinence • Exercise is perceived by the individual as an addiction • Desire to increase exercise constrained by life circumstances^*^

* *denotes symptoms which were, in at least one case, mentioned in a study in which some participants had a psychiatric diagnosis*.

We have grouped the identified symptoms into seven distinct categories: Body, Food, Negative Experiences, Positive Experiences, Control, Withdrawal Symptoms and Other Characteristics. In the case of Withdrawal Symptoms, all items in the category were explicitly listed as being withdrawal symptoms in the text. In all other cases, the categorization was made *post-hoc* by the authors.

### Symptoms Fitting DSM-5 Criteria for Gambling Disorder

In Table 3, we listed the 9 DSM-5 criteria for diagnosing gambling disorder, and the symptoms identified in this review which correspond to these criteria. The criterion for which parallels were most frequently found is “needs to gamble with increasing amounts of money in order to achieve the desired excitement.” In the case of excessive exercise, this is characterized by increasing exercise duration, in some cases specifically against the directions of a coach.

**Table 3:** DSM-5 criteria for gambling disorder, and suggested criteria for exercise addiction.

**DSM-5 criteria for gambling disorder**	**Symptoms identified in metareview**	**Articles mentioning this criterion**	**Suggested criteria for exercise addiction**
1. Needs to gamble with increasing amounts of money in order to achieve the desired excitement	• Coached athletes train beyond what is prescribed by trainer (7, 28). • Exercise volume has increased over time (5, 6, 8, 9, 24).	*n* = 2	1. Exercise volume has increased over time in order to avoid negative feelings of guilt or laziness
2. Is restless or irritable when attempting to cut down or stop gambling	• Withdrawal symptoms (for complete list see category in Table 2) (9, 21, 24, 26, 31).	*n* = 5	2. Negative affective response when exercise is reduced or sessions are missed/stopped
3. Has repeated unsuccessful efforts to control, cut back, or stop gambling	• The affected person feels unable to stop/ is unable despite attempts (5, 7, 9, 21, 23).	*n* = 5	3. Attempts to reduce exercise volume are feared and/or unsuccessful
4. Is often preoccupied with gambling (e.g., having persistent thoughts of reliving past gambling experiences, handicapping or planning the next venture, thinking of ways to get money with which to gamble).	• Thoughts constantly turning to exercise/rumination (20, 26).	*n* = 2	4. Is often preoccupied with exercise (e.g., having persistent thoughts of when and where next session will take place, planning training, thinking of ways to exercise during other activities)
5. Often gambles when feeling distressed (e.g., helpless, guilty, anxious, depressed).	• Exercise as a way to control/relieve negative mood and emotions (21, 23, 25, 26, 30)	*n* = 5	5. Exercise is used as a way to cope with negative life experiences or stressors
6. After losing money gambling, often returns another day to get even (“chasing” one's losses)	• -	-	6. Exercise is continued in spite of illness, injury or severe pain, at levels beyond rehabilitative training
7. Lies to family members, therapist, or others to conceal the extent of involvement with gambling	• Refusing to admit to, lying or minimizing behavior (8, 29)	*n* = 2	7. Lies about or minimizes time and intensity of exercise
8. Has jeopardized or lost a significant relationship, job, or educational or career opportunity because of gambling	• Exercise causes difficulties in carrying out work/study responsibilities, may lead to reducing working hours/leaving job/studies (5, 7, 20, 24, 28). • Exercise habits cause conflicts in social relationships (5, 7, 8, 22, 24, 28, 30).	*n* = 5 *n* = 6	8. Has jeopardized or lost a significant relationship, job, or educational or career opportunity because of exercise
9. Relies on others to provide money to relieve a desperate financial situation caused by gambling	-	-	9. Despite rational understanding of the negative physical and/or psychological burden of exercise habits, habits are continued.
-	-	-	10. Feeling of guilt when exercise is missed or reduced.

In two cases (“After losing money gambling, often returns another day to get even”; “Relies on others to provide money or relieve a desperate financial situation caused by gambling.”), no parallels in exercise behavior were identified.

In Table 3, we also presented initial suggestions for ten criteria to use in a clinical interview where excessive exercise appears to be a factor. These criteria reflect both the gambling disorder and substance-related disorder criteria of the DSM-5. This suggestion is a first approach to developing criteria based on empirical evidence drawn from the first meta-review of symptoms accompanying excessive exercise. It is neither exhaustive nor complete without further detailed screenings, but may serve as a guideline for clinical interviewing that aims to establish more about the nature of excessive exercise.

### Quality Assessment

The results of the quality assessment were presented in Figure 2. The majority of the studies were found to be of acceptable quality, with most criteria being fulfilled or at least partially fulfilled. Three studies (6, 36, 40) had mostly partially fulfilled or unfulfilled criteria. Notably, the issues of the researcher-participant relationship and the ethical issues considered in the study design were frequently categorized as being only partially or not at all fulfilled. As noted above, no studies were excluded on the grounds of poor quality. Consequently, the results of this meta-review include poor quality and non-peer-reviewed studies.

## Discussion

This meta-review provides, for the first time, a list of symptoms reported as arising in individuals who exercise excessively. Excessive exercise is reported to result in physical and psychological problems for those affected, and consequently may require treatment to improve mental and physical well-being. This review is intended to contribute to the categorization of excessive exercise as either a symptom cluster which accompanies other disorders, or a definable behavioral addiction. The results indicate that there is sufficient evidence to further explore the phenomenon in terms of a behavioral addiction. While our findings cannot be compared with a prospective study to identify disease history, combining results from a variety of narrative reviews and case studies enables us to obtain detailed information about the nature of this phenomenon.

### Fulfilling the DSM-5 Criteria for a Non-substance Related Addictive Disorder

A key question is whether the evidence gathered here indicates that exercise should indeed be considered as a form of behavioral addiction. Based on the findings of the meta-review, there is evidence that excessive exercise does indeed resemble gambling disorder, and hence that it could be considered a behavioral addiction. The symptoms identified represent a psychological (and sometimes physical) burden to the affected individuals which are comparable to those of the DSM-5 criteria for non-substance related disorders, and based on these, a similar list of diagnostic criteria has been established. The diagnostic criteria suggested here include an increase in exercise volume to avoid feeling lazy, an inability to reduce exercise volume, lying to family and friends about time spent exercising, and continuing despite illness or injury. These criteria resemble those of substance-related or gambling disorder, but are specifically based on experiences reported in the context of excessive exercise.

Importantly, it should be emphasized that a number of the symptoms identified were categorized as “positive experiences.” As with most addictive substances and behaviors, sport is often enjoyed by humans, and can, according to certain reports, lead to a “high” sensation (41). Seeking these positive experiences, to the degree that control over consumption or activity is lost, is sometimes cited as a feature of an addictive disorder. While not explicitly reflected in the DSM-5 criteria (5), it is a feature of the ICD-11 guidelines on disorders due to substance use (42). While the positive experiences become increasingly overshadowed by negative ones as an addiction to substances develops, certain positive elements can still remain (43). The same may be the case in excessive exercise.

Following our initial categorization, we have also sought to identify symptoms which appear to match the DSM-5 criteria for gambling disorder. Our reason for this was to present an initial example of whether exercise behaviors could fit an established framework of a behavioral addiction. By first creating an exhaustive list of symptoms, and only then searching to establish parallels with other addictive disorders (rather than slipping the word “exercise” into the DSM criteria *a priori*), we present empirical evidence for excessive exercise to be categorized as a behavioral addiction. To date, gambling disorder is the only behavioral addiction with established criteria in the DSM-5. However, gambling and exercise behaviors naturally differ greatly, and mapping one onto the other is not a viable approach to establishing a diagnosis. Our aim here was to present evidence that excessive exercise has characteristics understood, in other contexts, to constitute an addiction; the inclusion of “exercise addition” in the DSM framework would certainly entail the development of exercise specific criteria, as we have suggested with our examples here. However, this step must be preceded by a thorough investigation of the etiological process that underlies excessive exercise behavior, to avoid the pitfalls of reification that have affected work on other behavioral addictions (17).

Finally, criteria for “exercise addiction” will need to be precise enough to avoid automatically including highly motivated or elite athletes. While a number of studies seem to indicate that athletes (as opposed to fitness and recreational exercisers) report equally high or higher scores on exercise addiction questionnaires, compared to other exercisers, this findings have been received critically by several authors. Szabo et al. (11) summarize the quantitative data on this topic, and point out that high level athletes are very unlikely to have lost control of their behavior, or exercise beyond what is required to excel at their sport; they will also, by necessity, recognize the benefits of recovery to improve performance. In their study reporting that athletes have higher scores for exercise addiction, but also passion for sport, de la Vega et al. (44) suggest that athletes interpret exercise addiction questionnaires differently; for example, they may indeed be “unable” to reduce time spent exercising, but this is because they understand that completing a planned session is a part of attaining their goals, perhaps as instructed by a coach; it is not due to pathological compulsion. The lack of sensitivity of existing questionnaires, which may result in athletes being incorrectly characterized as at risk of addiction, is further discussed in the section Quantitative Measures below. A more precise screening instrument could, potentially, also contain items which reduce the overall score, such as “Are you a professional or performance athlete?”, and “Are you training for a specific event?”

### Eating Disorders

A potential confounder of our results is that a number of the studies included here represent respondents diagnosed with eating disorders. It has been suggested that excessive exercise habits are always “secondary” to an eating disorder. In other words, while exercise might still constitute a behavioral addiction, it would appear only in individuals with eating disorders (27). On this understanding, our results might be “contaminated” with symptoms that are really the result of an eating disorder. As noted above, the majority of symptoms in our exhaustive list were mentioned in studies with sufferers of eating disorders. However, twenty are not, and of those which are, it cannot conclusively be stated that they are fully explained by the eating disorder (some studies, for example, have samples which include individuals with and without eating disorders). It is also possible that in individuals who are addicted to exercise, disordered eating patterns emerge later, perhaps as a consequence of exercise-induced malnutrition (45). It is clear that excessive, perhaps compulsive exercising plays a role in a number of eating disorder types, and is engaged in by up to 50% of sufferers (46). Our results here, which include a study where a diagnosis of anorexia was an exclusion criterion (28), provide initial evidence that excessive exercise is more than just an eating disorder symptom.

### Quantitative Measures

In certain studies, quantitative methods were used to categorize or otherwise assess participants. Instruments used included the Exercise Addiction Inventory, the Exercise Dependence Scale, and the Exercise Dependence Questionnaire. As noted in the Introduction, these instruments are not based on diagnostic criteria for exercise addiction, but follow conceptual developed from addiction research in general. Due to the noted issues with varying prevalence estimates which have arisen following the use of these questionnaires, there have been concerns about their usefulness. A key aim of the current study was therefore the elaboration of criteria which are based solely on empirical reports of excessive exercise, an approach which has to date not been employed in the development of instruments assessing the risk of exercise addiction.

Our approach permits a valuable comparison of qualitative results with certain quantitative measures, allowing these to be given some context, based on the experiences of affected individuals. The category of “inconsistency” in Table 1 was introduced to illustrate when discrepancies seem to occur between the results of questionnaires and interview responses. Concretely, an inconsistency is an instance of a questionnaire and a qualitative questioning technique yielding different results with regards to an individual's risk of exercise addiction.

Johnston et al. (29) explicitly address this issue when describing their results:

“For example, Linda (categorized as a non-obligatory exerciser) stated: *I often think that if I…became ill or I had an injury that would prevent me from exercising, would be just the worst possible thing that could happen in my life!* At the other extreme, Deborah, a 77-year-old with arthritis whose sole exercise involved walking her dog, was classified as an obligatory exerciser.”

As the content of questionnaires in the field of excessive exercise have been the subject of debate (13), this is a useful indicator of the potential and limitations of these quick screening tools. As the previous example suggests, an individual who reports that they have to exercise and cannot reduce the volume may very well be carrying out a responsibility, in this case dog-walking. Developing questionnaires based on a systematic examination of excessive exercise habits and behaviors appears to be of importance.

### Implications

Classifying excessive exercising as a behavioral addiction will have implications for the therapeutic approaches employed to manage it. Primarily, if it is to be distinguished from other mental disorders such as eating disorders, the focus of therapeutic discussions will be substantially different. The aim of our initial clinical diagnostic criteria is to serve as a guide to the development of clinical and therapeutic approaches to excessive exercise. A status as an addictive disorder would, for example, suggest that attention might need to be paid to the positive reinforcing effects of exercise, with cessation of activity potentially negatively impacting mood in a manner that requires targeted treatment. Any physiological similarities to other addictive disorders (e.g., dopaminergic activity, reduced prefrontal cortical activity) may also guide treatment strategies by employing methods or pharmacotherapies used for other diagnoses.

### Limitations

We chose to focus only on qualitative research in this study. Consequently, many high-quality studies have been excluded because they report only quantitative results. While the aims of our study could only be met by employing qualitative data, we acknowledge that a large amount of the research into excessive exercise is not represented here.

By including studies that focus on excessive exercise, rather than other psychiatric conditions where excessive exercise habits happen to be present, we necessarily excluded a number of studies which may mention symptoms linked to excessive exercise (47, 48). We adopted this approach in order to ensure, as far as possible, that our symptom list contained findings that represent the literature on excessive exercise. In order to include as many case studies as possible, we opted to include papers, such as Letters to the Editor, which are not peer-reviewed.

We sought to provide as complete a list as possible of symptoms and characteristics that have been reported in association with excessive exercise. We established category headings in order to make this more user-friendly. However, we fully acknowledge that our categorizations may represent certain biases, and that certain readers may disagree with separating themes such as “Food” and “Body,” particularly in a context linked to eating disorders.

It must also be noted that in certain cases, authors have reported symptoms within the framework of a definition of addiction. Consequently, it is possible that certain symptoms have been described in a specific way, and other potentially underemphasized or overlooked, as the authors sought to match symptoms with a categorization of “addiction.” This approach necessarily influences the contents of our list, and must be borne in mind for its interpretation.

## Conclusion

This meta-review of studies suggests that a number of symptoms reported by individuals affected by excessive exercise resemble symptoms of other addictive disorders. The findings therefore indicate that there is sufficient evidence to further explore the phenomenon in terms of a behavioral addiction. The results further suggest that there is some inconsistency between risk for “exercise addiction” as measured by self-reports, and the presence of symptoms and diagnostic criteria reported in detailed clinical interviewing. While we conclude that the evidence suggests excessive exercise is likely to be a behavioral addiction, future studies must analyze the relevance of other co-occurring mental disorders, and employ more sensitive interviewing techniques on excessive exercise patterns.

## Data Availability Statement

The datasets analyzed in this article are not publicly available. Requests to access the datasets should be directed to Flora Colledge, flora.colledge@unibas.ch.

## Author Contributions

FC developed the research question, carried out the database search, and drafted the manuscript. RC replicated the database search and revised the manuscript. UB, AS, UP, MG, GW, and UL revised the manuscript. MW developed the research question, resolved differences regarding studies to be included, and revised the manuscript. All authors contributed to the article and approved the submitted version.

## Conflict of Interest

The authors declare that the research was conducted in the absence of any commercial or financial relationships that could be construed as a potential conflict of interest.
